# A Magnetic Flux Leakage and Magnetostrictive Guided Wave Hybrid Transducer for Detecting Bridge Cables

**DOI:** 10.3390/s120100518

**Published:** 2012-01-05

**Authors:** Jiang Xu, Xinjun Wu, Cheng Cheng, Anran Ben

**Affiliations:** State Engineering Research Center of Manufacturing Equipment Digitization, Huazhong University of Science and Technology, Wuhan 430074, China; E-Mails: xinjunwu@mail.hust.edu.cn (X.W.); 516873147@qq.com (C.C.); benyue2005@163.com (A.B.)

**Keywords:** hybrid transducer, magnetic flux leakage testing, guided wave, magnetostrictive effect, bridge cable, nondestructive testing, PACS 43.38.Ct, 43.35.Zc, 81.70.Ex

## Abstract

Condition assessment of cables has gained considerable attention for the bridge safety. A magnetic flux leakage and magnetostrictive guided wave hybrid transducer is provided to inspect bridge cables. The similarities and differences between the two methods are investigated. The hybrid transducer for bridge cables consists of an aluminum framework, climbing modules, embedded magnetizers and a ribbon coil. The static axial magnetic field provided by the magnetizers meets the needs of the magnetic flux leakage testing and the magnetostrictive guided wave testing. The magnetizers also provide the attraction for the climbing modules. In the magnetic flux leakage testing for the free length of cable, the coil induces the axial leakage magnetic field. In the magnetostrictive guided wave testing for the anchorage zone, the coil provides a pulse high power variational magnetic field for generating guided waves; the coil induces the magnetic field variation for receiving guided waves. The experimental results show that the transducer with the corresponding inspection system could be applied to detect the broken wires in the free length and in the anchorage zone of bridge cables.

## 1. Introduction

The common aspect of many long span bridges, such as suspension bridges, cable-stayed bridges and steel arch bridges, is that the cables are the primary load-carrying members [1]. The design life of these bridges is about 100 years, however the design life of cables is often less than 30 years. The inconsistency between the design life of bridges and cables is due to the fact that cables may be damaged due to fatigue and corrosion. The damaged cable can be replaced by a new cable, although it is costly. Furthermore, the service life of cables is often less than the design life, sometimes even less than 10 years. The main reasons are still fatigue and corrosion.

**Figure 1.**
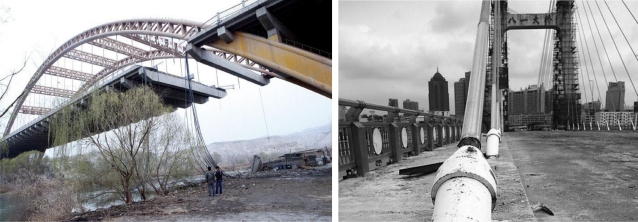
Two recent bridge failures in China.

The most important problem in the cable maintenance is the event of moisture intrusion. Moisture allows for the biological and chemical attack by making the materials easier to corrode. Although there are many precautionary measures implemented to protect the cable, the ingress of moisture is difficult to avoid when the sheathing is cracked. Water intruding through the crack is often accumulated in anchorage zones. The anchorage zone is vulnerable due to the local stress or notches created by the anchoring device. The anchorage zone has more severe corrosion problems than the free length [2]. Another reason for deterioration of cables is fatigue. The fatigue fractures often occur in the free length of cable. Corrosion and fractures have impacts on the strength of cables, which may have a significant influence on the cable's load-carrying capacity. Figure 1 shows two recent bridge failures owing to corrosion and fractures of the cables in China [3,4]. One hundred forty-four cables of the right bridge were replaced, which cost 80 million RMB (about 14 million US dollar). Therefore, the condition assessment of cables is very important to the bridge safety. On one hand, flaws can be detected early for avoiding serious accidents. On the other hand, only the flawed cables need to be replaced based on testing results and unnecessary costs can be avoided.

**Figure 2.**
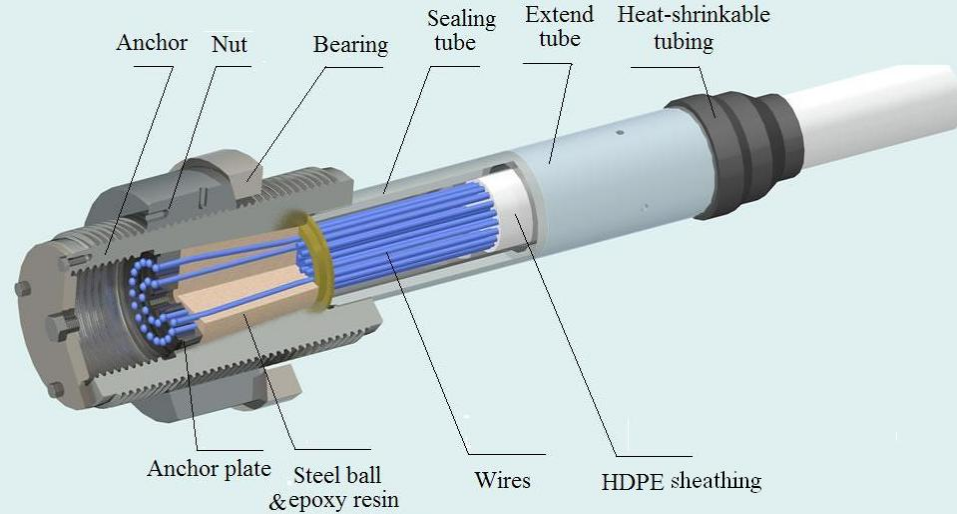
Schematic diagram of a parallel wire cable.

The parallel wire cable and the seven-wire strand cable are the most commonly used forms in the long span bridge. We will focus on the parallel wire cable in this study. Figure 2 shows the schematic of a parallel wire cable. There is high-density polyethylene (HDPE) sheathing on the free length of cable for corrosion protection. The thickness of sheathing is from several millimeters to tens of millimeters. The pipe near the anchor plate is typically filled with epoxy resin, and small steel balls. The rest of the pipe is often empty. Corrosion tests by Hamilton showed that corrosion was found inside the 7-wire strand without epoxy-coated in the interstitial spaces between wires, but little corrosion was found inside the filled strands [5]. His results demonstrated that the epoxy coating is helpful to avoid corrosion. The area filled with epoxy resin can also avoid corrosion. The anchorage zone mentioned in the following context limits in the unfilled epoxy-coated zone where corrosion often happens. The region is covered by the sealing tube, and the extend tube.

Various nondestructive testing (NDT) methods have been applied to inspect cables and wires, such as visual inspection [6,7], radiographic testing [8,9], magnetic flux leakage (MFL) testing [10,11], acoustic emission testing [12], vibration testing [13–15] and guided wave testing [16–21], especially magnetostrictive guided wave (MGW) testing [22–26]. The visual inspection method is the most commonly used method on cables. Only cable surfaces and accessible anchorage surfaces could be visually inspected. The use of radiography is costly and the traffic would be disturbed, although the interior structure of cables can be imaged. MFL testing has high detection accuracy in the cable. However, the localized measurement limits its application and only flaws in the free length of cable can be detected. It is impossible to detect the anchorage zone by MFL testing. Acoustic emission testing, as a dynamic and real-time inspection method, is low in efficiency for pre-existing flaws. Since guided waves can travel a long distance from the excitation position, MGW testing is suitable for inspecting the inaccessible zones. Although the method could be applied to detect flaws in the free length of cable, the detection accuracy is declined as the distance increases. In order to evaluate the well-being of cables, both the free length of cable and the anchorage zones need to be examined. Based on the requirement of the cable testing and the principles of MFL and MGW testing, a hybrid transducer for detecting bridge cables is provided in this study.

The organization of the paper is as follows. Section 2 is a brief introduction to the principles of MFL and MGW testing. The similarities and differences of the two methods are analyzed, and the main functions of the hybrid transducer are presented. The structure of the transducer is given in Section 3. Two functions are considered in designing each unit of the transducer. The experiment on a sample cable is provided in Section 4. The corresponding detection system is described. The testing process and the experimental results are provided. Conclusions of the paper are drawn in the last section.

## 2. Theoretical Backgrounds

### 2.1. Principle of MFL Testing

**Figure 3.**
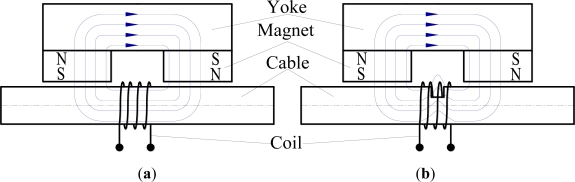
Principle of MFL testing. (**a**) Undamaged cable; (**b**) Cable with metal loss.

MFL testing is a magnetic based NDT method. The method is used to detect corrosion and cracks in ferromagnetic materials, such as pipelines, storage tanks, ropes and cables [10,27–29]. The basic principle of MFL testing is that the flux lines pass through the steel wires when a magnetic field is applied to the cable. At areas where corrosion or missing metal exists, the magnetic-field leaks from the wires. In an MFL tool, magnetic sensors are placed between the poles of the magnet to detect the leakage field. The signal of the leakage field is analyzed to identify the damaged areas and estimate the amount of metal loss. Thus, the transducer includes magnetizers and magnetic sensors. The magnetic-field can be produced by a permanent magnet yoke, or a solenoid with the direct current. The magnetic-field density needs to meet near saturation under the sensor. Figure 3 shows the principle of MFL testing. The permanent magnet yoke is used to produce the magnetic-field, and the coil is used to induce the leakage field.

### 2.2. Principle of MGW Testing

The guided wave technology is an effective NDT method [30–34]. As guided waves can travel a long distance from the excitation position, the technology can be applied to inspect the inaccessible region. The reflected wave will occur when the wave encounters corrosion or broken wires. The information of defects can be obtained by the echo wave. Several effects are applied to generate guided waves, such as the piezoelectric effect, the Lorenz force and the magnetostrictive effect.

**Figure 4.**
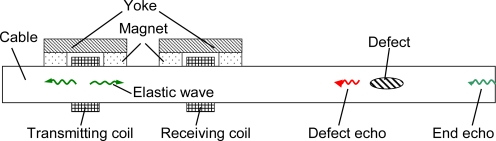
Principle of MGW testing.

The thickness of HDPE sheathing is up to tens millimeters on the cable surface. Removing sheathing is costly and unacceptable. Only MGW technique can generate and detect guided waves in the cable without removing the HDPE sheathing. The magnetostrictive effect (also known as the Joule effect) is that the shape of ferromagnetic materials will change when they are subjected to a magnetic-field [35]. The inverse magnetostrictive effect (also known as the Villari effect) is the change of the magnetic susceptibility of ferromagnetic materials when they are subjected to a mechanical stress [36]. MGW testing generating guided waves in cables is based on the Joule effect and detecting guided waves is based on the Villari effect. Figure 4 shows the principle of MGW testing for cables. Only the axisymmetric longitudinal mode guided wave is considered because generating the torsional mode wave in cables is difficult. To improve the coupling efficiency and avoid the second harmonic generation effect, the material under the transducer needs to be magnetized. The transduction efficiency increases, firstly, and then decreases with increasing the bias magnetic field density [37]. Thus, the transducer needs to provide a high power axial varying magnetic field and an appropriate axial static magnetic field to generate guided waves. To detect the reflected waves, the transducer should provide an appropriate axial bias magnetic field and induce the weak axial varying magnetic field.

### 2.3. Similarities and Differences of the Two Methods

As mentioned above, the similarities of the two NDT methods are that they both need an axial static magnetic field to magnetize the cables and a magnetic sensor to induce the axial varying magnetic field. The differences are that MFL testing requires a near saturated static magnetization state and MGW testing demands an appropriate static magnetization state. The operation frequency of MFL testing is lower than that of MGW testing. In addition, MGW testing needs the transducer to produce a high power axial varying magnetic field.

If only one transducer is employed as not only MFL sensor but also MGW transmitter and receiver, much work needs to be done. Firstly, the duplex is employed. One transducer can be used as the transmitter and the receiver of MGW testing. Secondly, the transducer includes a static magnetizer and the density of the magnetizer is suited to both MFL testing and MGW testing. Thirdly, the sensor includes a magnetic sensor. The sensor can be used to detect the magnetic-field variation due to the leakage field and the stress of guided waves. To detect flaws in the free length of cable by MFL testing, the transducer must move along the cable. It works at a continuous state. Owing to wave attenuation in cables, the transducer needs to get as closely as possible to the anchorage zone to ensure detection accuracy by MGW testing. Therefore, the transducer makes a demand of attaching a moving structure. In next section, the transducer is designed based on the above analysis.

## 3. Transducer Design

Based on the principles of MFL and MGW testing, the transducer for detecting cables was designed which consisted of several magnetizer units, a ribbon coil and a climbing mechanical structure.

### 3.1. Magnetizer

The magnetizer had three functions. First, it provided a near saturation static magnetic field for MFL testing. Second, it provided an appropriate axial bias magnetic field for MGW testing. Third, it provided attraction for the climbing mechanical structure.

**Figure 5.**
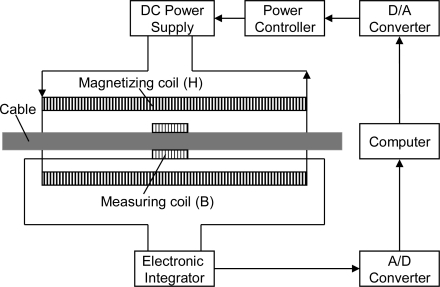
Schematic diagram of the experiment system for obtaining the magnetization curve of the cable.

**Figure 6.**
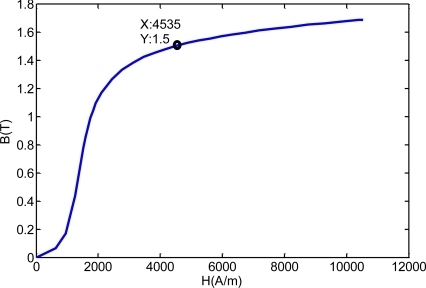
Magnetization curve of the cable.

Firstly, we studied the magnetic flux density to meet the needs of MFL testing. The magnetization curve of the cable was used to determine the magnetic flux density. The experiment was carried on a parallel wire cable with 163 × 7 mm wires, with 114 mm diameter, 7 m long, 8 mm thick HDPE sheathing. The experiment system for obtaining the magnetization curve is provided in Figure 5 [38]. The magnetization curve of the cable is shown in Figure 6. When the magnetic flux density of the cable was larger than 1.4 T, the cable was in the near saturation state. Considering the testing requirement of inner defects, the magnetizer should provide at least 1.5 T under the detector to satisfy the requirement of MFL testing. Due to the effect of the demagnetizing field, the magnetization curve of the cable with a different diameter needs to be modified.

**Figure 7.**
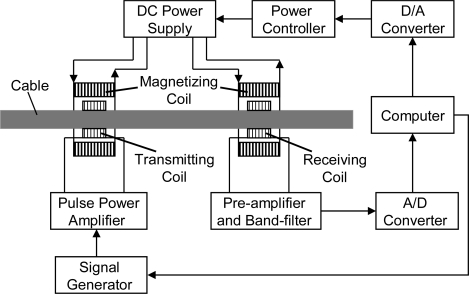
Schematic diagram of the experiment system for obtaining the relation of the transduction efficiency with the magnetic field density.

Secondly, we studied the magnetic flux density to meet the needs of MGW testing. The excited frequency of guided waves is about tens of kHz to hundreds of kHz. The initial oscillation zone was about tens of microns on the surface of the outer wires due to the skin effect. Hence, the magnetic flux density of the cable surface was concerned. The experiment was carried on the same cable as mentioned above. The experiment system for obtaining the relation of the transduction efficiency with the magnetic flux density is shown in Figure 7.

**Figure 8.**
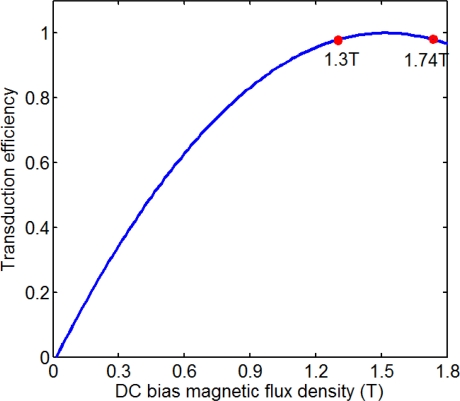
Normalized relation between the transduction efficiency and the magnetic flux density.

The transmitter included two coils; one provided the bias magnetic field and the other provided the high power varying magnetic field. The receiver also included two coils; one provided the bias magnetic field and the other induced the change of the magnetic field. The directly passing signal was applied to evaluate the transduction efficiency. A hall sensor was employed to detect the magnetic strength near the surface of the cable. The magnetic flux density was obtained from the magnetization curve. The optimum frequency of the tone burst was 80 kHz, and the first longitudinal mode guided waves (L(0,1)) was produced. The normalized relation of the transduction efficiency with the magnetic flux density is shown in Figure 8. We made the assumption that the transduction efficiency should be 98% maximum. The magnetic flux density of the cable was about from 1.3 T to 1.74 T corresponding to the assumption. The relation of the transduction efficiency with the magnetic flux density is different in the parallel wire cable with a different diameter. Therefore, the relation should be renewed in the cable with a different diameter.

The cable under the transducer was magnetized to 1.5 T to 1.74 T which satisfied both MFL testing and MGW testing demands. The attraction of magnetizer would be obtained after the structure of the magnetizer was determined. The magnetic field could be created by coils, or permanent magnets. Rare-earth magnets, neodymium-iron-boron magnets, were employed to provide the static magnetic field. Because the permanent magnets did not require the additional power supply which was suited for the field application. The magnetizer included permanent magnets and the yoke icon, and was modularized for the field application. By adjusting the number of modules, the magnetizer could apply to cables with different diameters.

### 3.2. Coil

The coil had three functions. First, it induced the changing magnetic-field due to the leakage field of defects (below 1 kHz); second, it provided a pulse high power varying magnetic field for MGW testing; third, it induced the various magnetic-field due to the stress of guided waves (up to tens of kHz). These magnetic-fields were axial. The solenoid, which could produce or induce the axial magnetic field, was appropriate for this application. A ribbon coil was employed for field application. The width of coil was about 50 mm, and the turn of coil was forty. The length of the coil was changed with the diameter of cables.

### 3.3. Climbing Structure and Overall Design of the Transducer

**Figure 9.**
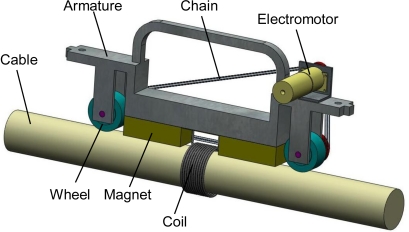
Structure of the climbing module.

**Figure 10.**
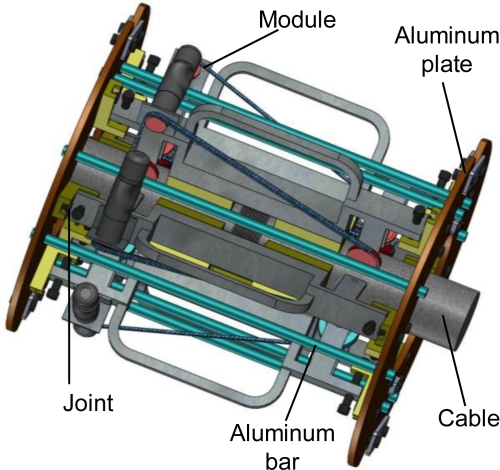
Structure of the transducer.

The climbing structure consisted of a framework of aluminum and several climbing modules. According to the cable diameter, the number of modules was selected to form the climbing structure. The climbing module was made up of a magnetizer, two wheels and a drive mechanism as shown in Figure 9. As mentioned in Section 3.1, the magnetic force would be produced between the permanent magnet and the cable. Wheels were made of synthetic rubber to increase the friction. The wheels were driven by an electromotor and a chain. Three factors should be considered by choosing the number of modules: the cable diameter, the attraction and the magnetization. Based on the above analysis, the structure of the transducer is shown in Figure 10.

## 4. Applications

### 4.1. Inspection System and Testing Process

**Figure 11.**
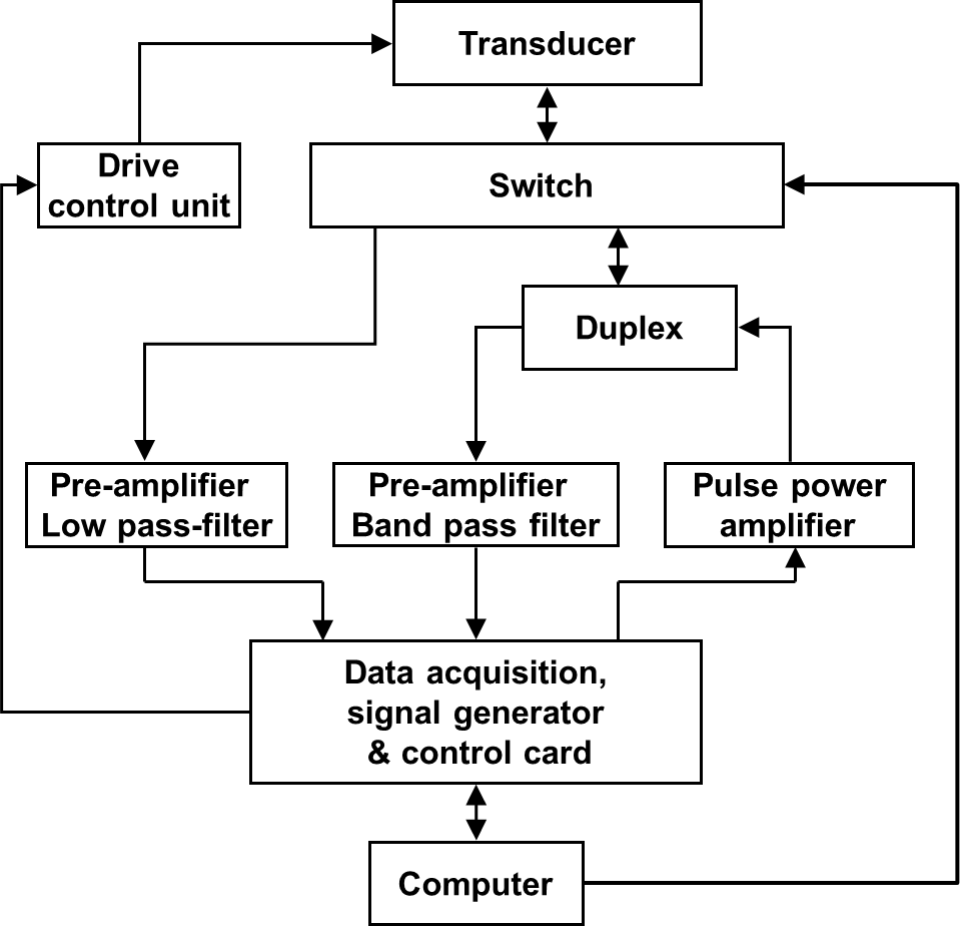
Schematic diagram of the corresponding inspection system for cables.

The corresponding inspection system for cables is shown in Figure 11.

The testing process is as follow:

Step 1: The transducer is installed on the cable.
Step 2: The anchorage zone of the near end is detected by MGW testing. The pulse power amplifier connects the transducer by the switch; the signal generator produces the tone burst to the pulse power amplifier; the high-power tone burst is input to the coil. Based on the Joule effect, guided waves are generated in the cable; based on the Villari effect, the reflections from the near end anchorage zone are obtained by the duplex; then the data passing the pre-amplifier with band pass filter are digitized to the computer.
Step 3: the free length of cable was scanned by MFL testing. The pre-amplifier with low pass filter connects by the switch of the coil; the motor is controlled to move along the cable to detect flaws in the free length of cable; the detection data passing the pre-amplifier with low pass filter are digitized to the computer.
Step 4: When the transducer is close to the other end of the cable, the motor stop and the Step 2 is repeated to detect the anchorage zone of the other end by MGW testing.

### 4.2. Experiments on a Sample Cable

**Figure 12.**
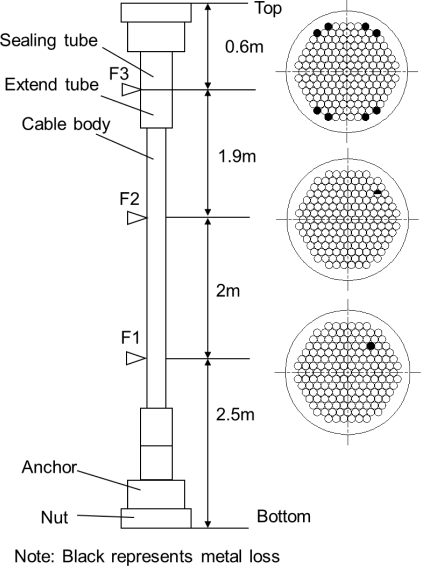
Schematic diagram of the sample cable.

The transducer was employed to detect a sample cable. The parallel wire cable was customized by Liuzhou OVM machinery Co. Ltd. who was one of the largest manufacturers of bridge cables in China. The sample was a PES7-163 cable, with 114 mm diameter, Φ7 mm × 163 wires (Φ7 mm steel wire, 1,670 MPa grade), 8 mm thick HDPE sheathing. The sample did not have cement grout in the sealing tube and the extend tube. There were three artificial defects in the cable as shown in Figure 12. The first defect was denoted as F1 which was one broken wire flaw in the free length of cable. The second defect was denoted as F2 which was a half broken wire flaw in the free length of cable. The third defect, coated by the steel tube, was denoted as F3 which was the flaw with eight broken wires in the anchorage zone. Here, the standard of the wire ropes (ISO 4309:2010 Cranes—Wire ropes—Care and maintenance, inspection and discard) was referred. If the number of broken wires was about 5% of the total wires, the bridge cable must be scrapped. Therefore, the number of broken wires in the sample cable was eight, which was about 5% of the total wires.

**Figure 13.**
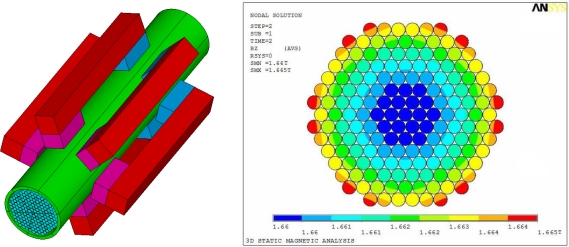
FE model and simulated results of the cable.

**Figure 14.**
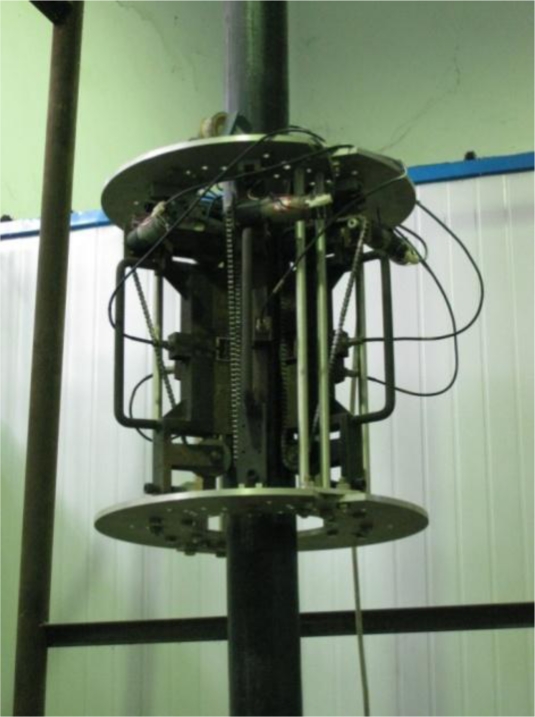
Prototype of the transducer detecting the sample cable in the laboratory.

The transducer consisted of six climbing modules, a coil and a framework. A commercial finite element (FE) software ANSYS was employed to study the magnetic flux density of the cable and the attraction between the transducer and the cable. The attraction of the transducer was about 3600 N; the adhesion coefficient was about 0.3; the weight of the transducer was about 85 kg. The FE results showed that the maximum magnetic flux density at the cable surface and the cable center were 1.665 T and 1.66 T, respectively, as shown in Figure 13. The cable under the transducer was magnetized to 1.5–1.74 T, which satisfied both MFL testing and MGW testing demands as mentioned in Section 3.1. Hence, the magnetic flux density was able to satisfy the requirements of MFL and MGW testing. The transducer positioning on the cable in the laboratory is shown in Figure 14.

**Figure 15.**
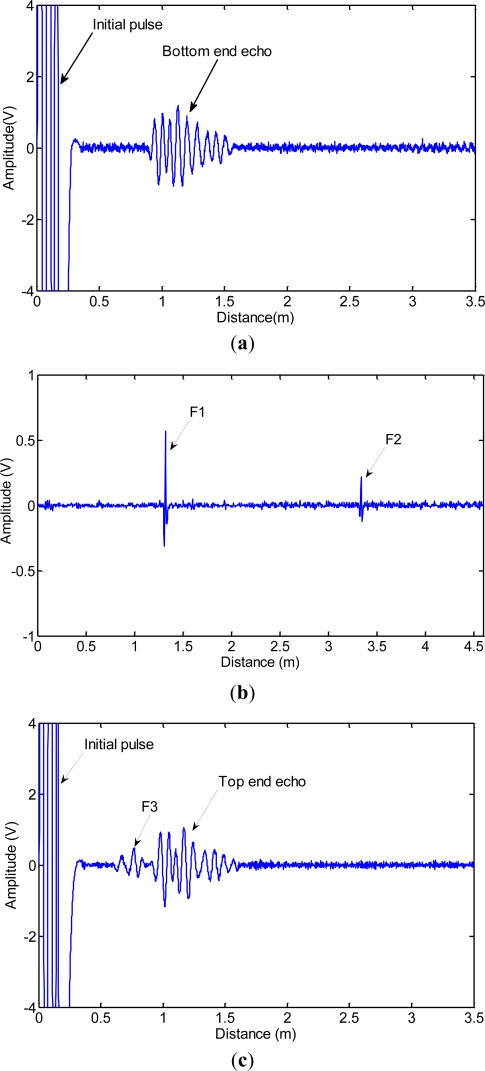
Detection data of the sample cable. (**a**) Data obtained by MGW testing when the transducer stopped at 1.2 m away from the bottom; (**b**) Data obtained by MFL testing when the transducer moved from 1.2 m to 5.8 m away from the bottom; (**c**) Data obtained by MGW testing when the transducer stopped at 1.2 m away from the top.

The testing process and the results are as follows. First, the transducer was installed at 1.2 m away from the cable bottom. The bottom anchorage zone of the sample cable was detected by MGW testing. The optimum frequency of the tone burst was 80 kHz with three cycles, and the first longitudinal mode guided wave (L(0,1)) was produced. The data is shown in Figure 15(a). The first signal was the initial electromagnetic pulse. The second signal was the echo from the filled epoxy-coated strands and the cable bottom. The echoes of F1 and F2 could not be observed in Figure 15(a). Second, the free length of cable was detected by MFL testing from 1.2 m away from the cable bottom to 1.2 m away from the cable top; the data is shown in Figure 15(b). The peak-peak value of F1 was 0.88 V and the peak-peak value of F2 was 0.34 V. The locations of detected flaws, F1 and F2, were about 2.518 m and 4.505 m from the bottom of the cable, respectively. Lastly, the transducer stopped at 1.2 m away from the cable top. The top anchorage zone of the sample cable was detected by MGW testing; the data is shown in Figure 15(c). The location of detected flaw, F3, was about 0.593 m from the top of the cable. The positioning error of F3 was about 1.2%. Likewise, the echoes of F1 and F2 could not be observed in Figure 15(c). The results indicated that F1 and F2 were too small to be detected by MGW testing.

## 5. Conclusions

A combined magnetic flux leakage and magnetostrictive guided wave transducer for detecting the bridge cables is provided in this study. The transducer consists of several magnetizer units, a ribbon coil and a climbing mechanical structure. The transducer could be used to detect broken wires in the free length of cable using MFL testing and in the anchorage zone using MGW testing. The detection accuracy of guided waves in the anchorage zone is less than MFL testing in the free length of cable. The hybrid transducer provides more information to evaluate the health of the cables than the simple transducer. In addition, the transducer could be used in other similar occasions, such as ropes and pipelines.

The research will be continued to determine the minimum detectable number of broken wires and the longest detectable range by using this transducer in future work. The relation between the amount of broken wires and the amplitude of the defect signals will also be studied.
